# Book Review

**DOI:** 10.1155/S1463924682000121

**Published:** 1982


### Book Review

### Personal Computers in Chemistry

Edited by P. Lykos (Wiley, Chichester, UK and New York, 1981), pp. 262+xi. £14·75 and $36·60. ISBN 0-471-08508-1.

This book contains a number of papers originally presented at a symposium 'Chemists and computers: now one on one' held at the National Meeting of the American Chemical Society in Washington, D.C., in August 1979. A total of 20 papers have been selected by the editor from more than 40 that were given at the symposium.

The first five papers describe the use of personal computers to acquire data from, and to control, single instruments. The next four describe the connection, via star networks, of several instruments to a central processor, which in two instances is a fairly substantial minicomputer. These are followed by two papers on graphics techniques, and five papers on various aspects of teaching, including computer-assisted learning. One of these describes a talking microcomputer built from a Zilog MCZ-1/20 and a Votrax model VSK voice synthesizer with the objective of helping blind students in chemistry laboratories. Another describes the transfer of the University of Illinois' PLATO system, which was developed for a system comprising 1000 terminals linked to a dual CDC-CYBER, to an 8080-based microcomputer environment. The book ends with four miscellaneous papers: Macero *et al.* describe, with considerable practical detail, an approach to machine independence based on the S-100 bus and the CP/M operating system; Schilling describes the use of an 8008-based microcomputer as an intelligent remote terminal to an IBM-370; Woodward writes briefly on an interactive laboratory data system; and the book finishes with a paper by Neece and Ostlund who describe a prototype simple multi-microprocessor based on a Z-80 processor and the S-100 bus.

This book is reproduced directly from the authors' typescripts, but the overall quality of production is good and there are few errors in the texts. As one would expect, the quality of the papers is variable, some are trivial and superficial but the majority are practically oriented and should prove useful, both to the novice who wants to find out how to get started, and to the more experienced worker who wants to know how others have tackled their problems. The non-systematic approach makes this book unsuitable as a textbook for students.

The index is virtually useless and gives the impression that it was compiled by a non-scientist with absolutely no knowledge of computing. Among its entries are: Analograte*s* [*sic*] of 100 MHz, Fast kinetic, High pressure mixing, Kirchoff equation, Link edited, Nanosecond, POKED and Polish string (and this is only a sample). Where sensible entries do exist many references in the text have been overlooked. Rather unusually, authors are identified only by name although in some cases it is possible to deduce where they work.

**D. G. Porter**

### Japanese Journal of Clinical Laboratory Automation

*JJCLA published the following articles in its October 1981 issue (Vol. 6, No. 4):*

'Man's real worth in the unmanned automated laboratory' (*Yasuhiro Ohba*)

'Examination of blood cell distribution with the ADC 500 spinner' (*Toshiyuki Akiyama*)

'A trial apparatus for automated blood coagulation profiling and data-processing' (*Nobumichi Takeishi*)

'Determination of erythrocyte size in hepato-biliary disorders using the MICROX automated cell analyser' (*Isamu Takeda*)

'The appearance of abnormal patterns in Hemalog D and their analysis' (*Jun Aoki*)

'Determination of iron assay in serum by Ferro Chem (Model 3050) II—effect by administration of iron' (*Miyoko Saito*)

'Automatic analysis of blood pyruvate kinase (PK) and its clinical application' (*Tetsuo Okanishi*)

'Application of immobilized enzymes to Olympus ACA6000R' (*Yoshitaka Morishita*)

'An automated method for measurement of p-aminobenzoic acid in the pancreatic exocrine function (PFD) test' (*Shigeru Iyama*)

'Therapeutic drug monitoring in the National Cardiovascular Center' (*Shigeki Ohgitani*)

'Enzyme immuno inhibition for the determination of CK-MB: application for ABBOTT-VP' (*Kanji Morita*)

'Evaluation of electrode potentiometry for automation of clinical microbiology' (*Michio Tanaka*)

'Evaluation of automated microbiology system of MS-2 and its application for MIC determination' (*Sachiko Eguchi*)

'Evaluation of Autobac MTS and MS-2 for screening of significant bacteria' (*Sachiko Satake*)

'The clinical laboratory system at Shimonoseki Ishikai Hospital' (*Setsuko Onizuka*)

'Development of "MAULIS"' (*Yasuhiko Fukada*)

'Report form of a clinical laboratory system' (*Mariko Motoi*)

'Voluntary developed system and personal data check method' (*Akira Nakamura*)

'On the modes of computer aided admission of test requests to the clinical laboratory department' (*Jiro Harada*)

'Total systematization of a clinical laboratory system (IV). The problems and their solutions at our clinical laboratory' (*Tatsuo Okishio*)

'Total systematization of a clinical laboratory system (V). Clinical laboratory test result inquiring system of ward patients' (*Takuro Morii*)

'Computerization of a laboratory data control system' (*Fumio Funatani*)

'The evaluation of cholesterol reagent with the new type cholesterol oxidase' (*Shigeru Yanai*)

